# Acupuncture reduced the medical expenditure in migraine patients: Real-world data of a 10-year national cohort study

**DOI:** 10.1097/MD.0000000000021345

**Published:** 2020-08-07

**Authors:** Sheng-Ta Tsai, Chun-Hung Tseng, Mei-Chen Lin, Hsien-Yin Liao, Boon-Khai Teoh, Shao San, Chon-Haw Tsai, Hung-Yu Huang, Yi-Wen Lin

**Affiliations:** a Department of Neurology, China Medical University Hospital; b Graduate Institute of Acupuncture Science, College of Chinese Medicine, China Medical University; c School of Medicine, College of Medicine, China Medical University; d Management Office for Health Data (DryLab), Clinical Trial Research Center (CTC); e Department of Acupuncture, China Medical University Hospital; f Department of Anesthesiology, China Medical University Hospital; g Chinese Medicine Research Center, China Medical University, Taichung, Taiwan.

**Keywords:** acupuncture, economic, health care system, medical expenditure, migraine, real world

## Abstract

**Objectives:**

According to the data of Organisation for Economic Cooperation and Development, almost all the countries got increased medical expenditures in these years. Among the diseases, migraine is a condition that affects predominantly young and middle-aged people. It results in great economic losses. So we perform this research to investigate the acupuncture effect of reducing medical expenditure and medical resources use.

**Perspective:**

Acupuncture is a non-pharmacologic treatment and it became popular in recent years. In Taiwan, about 13% migraine patients visited acupuncture doctor. We hypothesized that the acupuncture had the additional effect than the medical treatment.

**Setting:**

We analysed the economic cost and medical visits in the real word.

**Methods:**

We used national cohort data from Taiwan, retrospectively gathered between 2000 and 2010. We selected newly diagnosed migraine patients who were diagnosed by registered neurologists formally licensed by the Taiwan Neurological Society. We divided these patients into two groups: with and without acupuncture treatment. The main outcome was medical expenditures and visits within 1 year after acupuncture.

**Results:**

In migraine patients who received acupuncture treatment, medical expenditures on emergency care and hospitalization were significantly lower than the group without acupuncture treatment.

**Conclusion:**

According to our real-world data, acupuncture can reduce the medical expenditure in migraine patients within 1 year after diagnosis. For the health policy maker, it is cost effective to encourage combining acupuncture and western medicine to treat migraine patients. For the doctors in routine clinical practice, who may consider to consult acupuncture doctors to deal with the migraine patients together.

## 1 Introduction

According to the data of Organisation for Economic Cooperation and Development, almost all the countries got increased medical expenditures in these years. Among the diseases, migraine occurs with the highest prevalence between the ages of 25 and 55 years, potentially the most productive period of life.^[1]^ Migraine carries a significant economic burden in several countries.^[2–7]^ In 2016, globally about 1.04 billion (95% CI 1·00–1·09) people had migraine and migraine caused 45.1 million (95% CI 29·0–62·8) years of life lived with disability globally.^[8]^ In Taiwan, the prevalence of migraine is approximately 9.1% (female = 14.4%, male = 4.5%)^[9]^ and also causes numerous economic loss.^[10]^ Some migraine patients visited our clinics and told us that their migraine improved a lot by seeking acupuncture therapy. They had less emergency department visits due to severe headache, and which saved both their time and money. As a result, we retrospectively collected the data from our National Health Insurance Research Database, a real-world clinical data in Taiwan, to analyse the acupuncture effect among migraine patients, by comparing the numbers of emergency visits, hospitalization, and total medical expenditures.

## 2 Materials and methods

### 2.1 Study design and data source

The National Health Insurance Research Database (NHIRD) was established by the Taiwanese government and includes historical outpatient, hospitalization, emergency care, and medication information on each insurant. At present, the database covers more than 99% of Taiwanese citizens. To emphasize the protection of privacy, patient identification numbers were encrypted before the database was released by the government.

We conducted this study by searching the Longitudinal Health Insurance Database 2000, which is based on the NHIRD and includes 1 million randomly selected subjects from the original database, about 1/23 of the whole population (with the same age and sex distribution). The Longitudinal Health Insurance Database 2000 also included the diagnostic history of the Chinese herbal medicine department. The historical diagnoses are coded according to the International Classification of Disease, Ninth Revision, Clinical Modification (ICD-9-CM). The Research Ethics Committee of China Medical University and Hospital in Taiwan approved the study (CMUH-104-REC2-115-R3).

### 2.2 Study subjects

**Figure 1:**
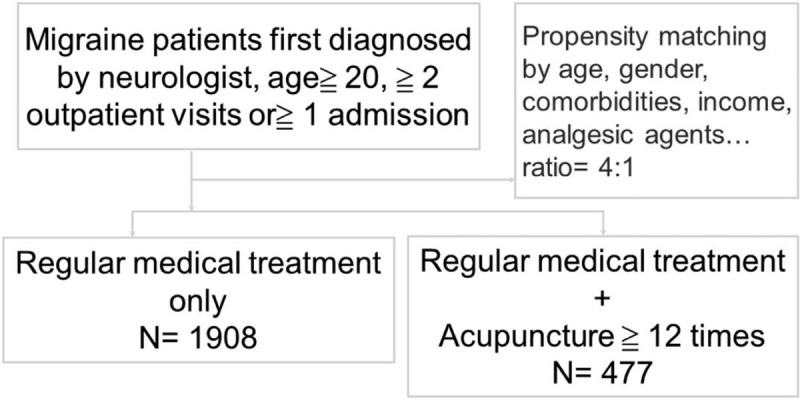
Research algorism in our study.

Eligible study subjects were patients with migraine (ICD-9-CM: 346) newly diagnosed by a neurologist and with at least two outpatient or one inpatient visit. The case group was comprised of patients who agreed to undergo acupuncture treatment greater than or equal to 12 times (2 packages) after migraine diagnosis; control group were 4:1 propensity score matched by demographic factors, comorbidities, migraine treatment drugs and frequency of migraine outpatients visits with case group (see Fig. 1).

This 4:1 ratio was according to their propensity score through nearest neighbor matching, initially to the eighth digit and then as required to the first digit. Therefore, matches were first made within a caliper width of 0.0000001, and then the caliper width was increased for unmatched cases to 0.1. We reconsidered the matching criteria and performed a rematch (greedy algorithm). For each patient with acupuncture treatment, the corresponding comparisons were selected based on the nearest propensity score.

We did this propensity score matching to reduce the confounding factors. The comorbidities of hypertension, diabetes mellitus, etc., are highly related to the medical expenditure^[11]^ and medical visits.^[12,13]^ According to the previous research,^[14,15]^ the migraine severity and acute medication use are associated with higher all-cause health care costs for patients with migraine. So we matched the two groups of patients by migraine acute treatment drugs and frequency of outpatients visits to control these confounding factors.

About the sample size calculation, we used the R software, package: epiDisplay (Epidemiological calculator, author: Virasakdi Chongsuvivatwong), under the condition of Alpha = 0.05, power = 0.9, n2/ n1 = 4 (by propensity score). The minimal required sample size was 112 patients in “acupuncture” group, and 445 patients in “medical treatment only” group. Our actual patient number is 477 and 1908 respectively, four times larger than the minimal requirement number.

### 2.3 Types of acupuncture used in this study

Eligible acupuncture types included manual acupuncture of the Traditional Chinese Medicine type (B41, B42, B45, B46, B80-B84, B90-B94, P27041, P31103, P32103, and P33031) and electro acupuncture (B43, B44, B86–89, and P33032).

### 2.4 Primary outcome and covariates

In this study, we aimed to investigate migraine-related medical expenditures and the number of visits for emergency care and hospitalization for migraine. The covariates included a history of diabetes mellitus (ICD-9-CM: 250), hyperlipidemia (ICD-9-CM: 272), mental disorders (ICD-9-CM: 290-319), epilepsy (ICD-9-CM: 345), hypertension (ICD-9-CM: 401–405), ischemic heart disease (ICD-9-CM: 410–414), congestive heart failure (ICD-9-CM: 428.0), osteoporosis (ICD-9-CM: 733.0, 733.1), stroke (ICD-9-CM: 430–438), asthma (ICD-9-CM: 493), and non-infectious kidney disease (ICD-9-CM: 580–589), as comorbidities. The interventional drugs discussed in this study came from the Taiwan migraine treatment guidelines,^[16]^ which are followed by most neurologists. These drugs were Sumatriptan [ATC (Anatomical Therapeutic Chemical) code: N02CC01], Rizatriptan (ATC code: N02CC04), Ergotamine (ATC code: N02CA52), Caffeine (ATC code: R05X, N02BE54, R01BA51, R05DA, N02BE51, N02BE71, R06AA52, R05FA02, N06BC, R06AK, N02CA52, G03BA, R06AK, M03BB53, R06AE55, M03BC51, M03BB03), Acetaminophen (ATC code: N02BE01, M03BB52, N02BE01, M03BC51, M03BA02, M03BB53, N02AX52, M03BA52), Ibuprofen (ATC code: N02CC01), Naproxen (ATC code: M01AE02), Diclofenac (ATC code: M01AB05), Celecoxib (ATC code: M01AH01), and Etoricoxib (ATC code: M01AH05). Age, gender, geographic region, income, and urbanization were the demographics-adjusted factors in this study.

### 2.5 Statistical analyses

To test for significant differences in demographic factors, comorbidities, and medications between migraine patients with or without acupuncture, a chi-square test was used; the mean age, frequency of visits, and medical expenditures on emergency care and hospitalization in the acupuncture and non-acupuncture groups were compared by an unpaired *t*-test. The statistical analysis was conducted with a type I error rate of α = 0.05 using the statistical software package SAS, version 9.4 (SAS Institute, Inc, Cary, NC).

## 3 Results

We enrolled a total of 2385 migraine patients (Table 1), including 477 with acupuncture treatment (case) and 1908 without acupuncture treatment (control). Approximately 75% were female, and the mean ages were 44.36 and 44.91 years in the case and control groups, respectively. After propensity score matching, there was no significant difference in demographic factors, comorbidities, frequency of migraine outpatient visits, or medication usage. Considering the type of acupuncture used among patients with acupuncture treatment, 74.4% only accepted manual acupuncture, 1.3% only accepted electro acupuncture, and 24.3% underwent a combination of manual acupuncture and electro acupuncture.

**Table 1:**
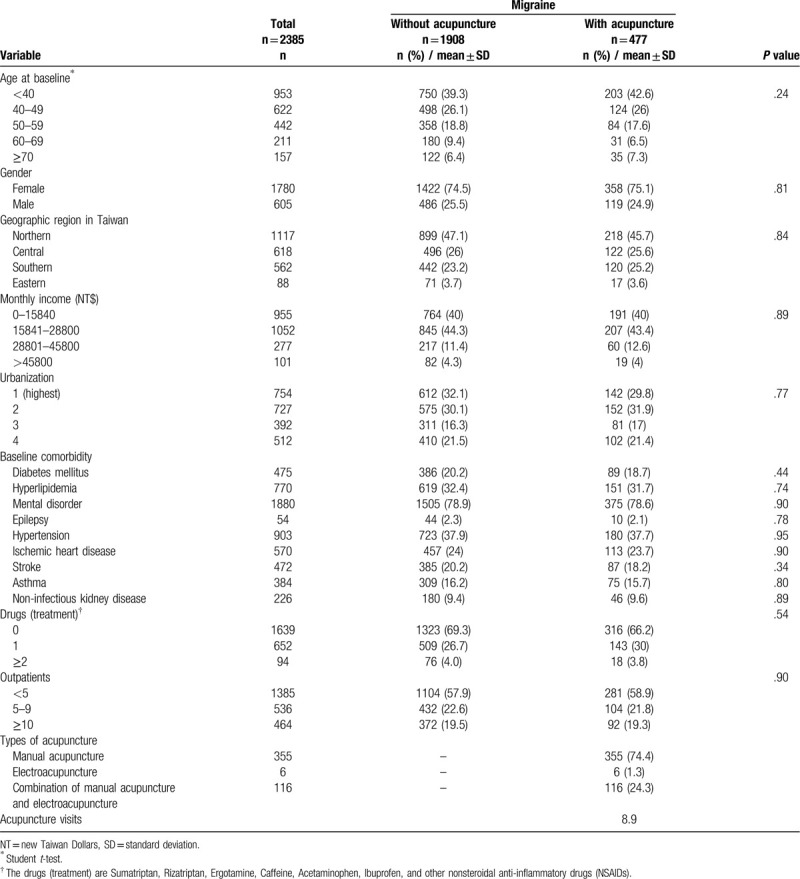
Demographic characteristics and comorbidities in patients newly diagnosed with migraine with or without acupuncture in Taiwan from 2000 to 2010.

Medical expenditures on emergency care (*P* = .01) and hospitalization (*P* = .01) were significantly lower in patients who underwent acupuncture treatment (see Table 2).

**Table 2:**
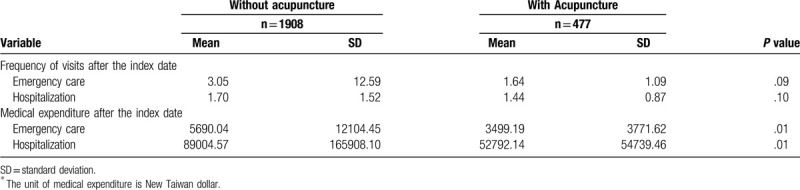
Medical visits and ^∗^expenditures in 1 year with and without acupuncture.

When stratified by medication usage (Table 3), migraine patients with more than one type of drug treatment combined with acupuncture had significant lower frequency of hospitalization (*P* = .03) and emergency care expenditure (*P* = .01) within one year. Which result may indicate that the severe migraine patients get more benefit from the combined acupuncture treatment.

**Table 3:**
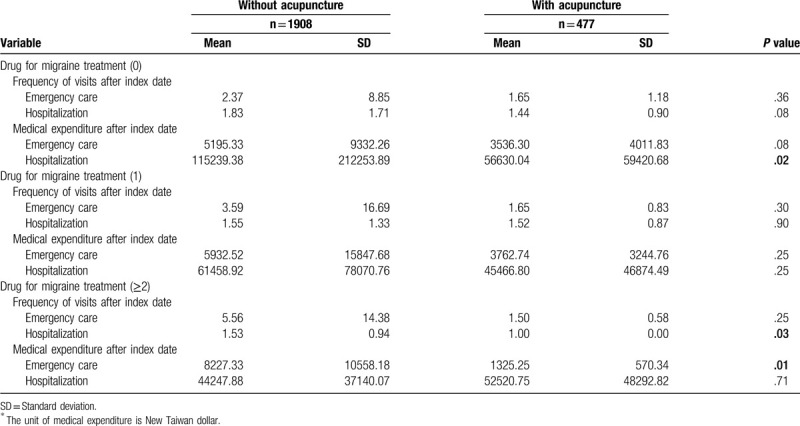
Medical visits and ^∗^expenditure of migraine, stratified by analgesic drugs.

## 4 Discussion

Acupuncture is a complementary and alternative medicine therapy in use for migraine. In the 2016 Cochrane Library,^[17]^ the authors concluded that adding acupuncture to symptomatic treatment of migraine attacks reduces the frequency of headaches. Thus, acupuncture can be considered a treatment option for patients willing to undergo this treatment. Eleven years ago, a randomized, controlled trial held in Germany found that acupuncture is cost effective in patients with headache.^[18]^ Two years ago, the group in Czech Republic published their randomized controlled trial, specified to investigate the cost effectiveness about acupuncture in “migraine” patients.^[19]^ Not only European countries demonstrated the efficacy of acupuncture, the team from Beijing did the randomized controlled trial and found that true acupuncture plus placebo is better than the group of sham acupuncture plus flunarizine.^[20]^ The Taiwanese team focused on the chronic migraine and found the acupuncture group got better improvement than the Topiramate group.^[21]^ And the adverse events were significantly lower in the acupuncture group (6%) than the Topiramate group (66%). Our real world data analysis is consistent to the finding of above several randomized control trials that acupuncture is cost effective in treating migraine patients.

The strength of this study was that it was the real world data reflecting the clinical practice and the real medical expenditure in Taiwan. Our study included a large number of patients to lessen the effects of minor confounding factors. The Taiwan Health Insurance Program has covered approximately 99% of the population since 1995, and a growing body of articles has confirmed the validity of the dataset.^[22]^ Instead of the prospective study, our retrospective analysis of the real world data supports the value of acupuncture treatment from the other side. The acupuncture add-on therapy can reduce the medical expenditure of migraine patients, and reduce the hospitalization in severe migraine patients.

The first limitation of our study is the retrospective design. There are some common threats to internal validity in retrospective studies.^[23]^ For example, the two groups weren’t randomly assigned. There was some existing selection bias. And there are another events, other than the intervention, influence the outcome. As we had mentioned in the section of “material and methods”, the comorbidities and disease severity are highly related to the medical expenditure and medical visits.^[11–15]^ The previous study using the same national cohort (NHIRD in Taiwan) also told us that age, income, and urbanization would significantly influence the medical expenditure.^[24,25]^ So we controlled these factors by the propensity matching (Table 1), trying our best to reduce their confounding effects. But there are still some other factors that we cannot control, for example, some social and behavior factors (influence the patients to choose acupuncture treatment).^[26]^

About the propensity score, in intervention trials, only randomization guarantees equal distributions of all known and unknown patient characteristics between an intervention group and a control group and enables causal statements on treatment effects.^[27]^ The propensity score is a widely used tool for non-randomized trials to analyze with multiple regression models. And the previous research had confirmed the power of this tool.^[28–31]^

The second limitation was the diagnosis of migraine. We could not select patients by the International Classification of Headache Disorders 3rd edition criteria^[32]^ because of lack of detail headache history. And we enrolled both chronic migraine (≥15 headache days/month) and episodic migraine (<15 headache days/month). The previous studies told us that chronic migraine had greater medical expenditure than the episodic migraine.^[33,34]^ We attempted to increase the precision of diagnosis by selecting migraine patients who were first diagnosed with migraine by a certificated neurologist. Most of our certificated neurologists adhere to the Chinese form of International Classification of Disease, Ninth Revision, Clinical Modification criteria translated by Taiwan Headache Society.

The third limitation was the different severity of migraine patients.^[35]^ We matched the two groups by the number of analgesic drugs (acetaminophen, triptans, nonsteroidal anti-inflammatory drugs, and others) and the number of outpatient department visits (generally more severe patients visited the clinic more often). We did not match the patients for migraine preventive medication (propranolol, flunarizine, etc.) use because of the low prescription rates and poor drug adherence.^[36–38]^

The forth limitation was the overlapping of neck myofascial pain and migraine.^[39]^ Clinically, several migraine patients complained not only headache but also neck pain or tenderness. We speculated, on the basis of previous research, that the relief of neck pain improved the patients’ symptoms of migraine and prevented further migraine attacks.^[40–52]^

**Figure 2:**
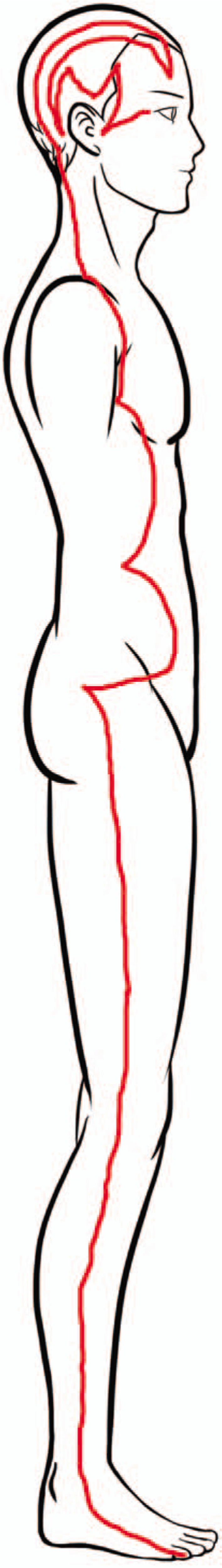
Brief illustration of Foot Shaoyang meridian (= Gallbladder meridian, GB meridian).

**Figure 3:**
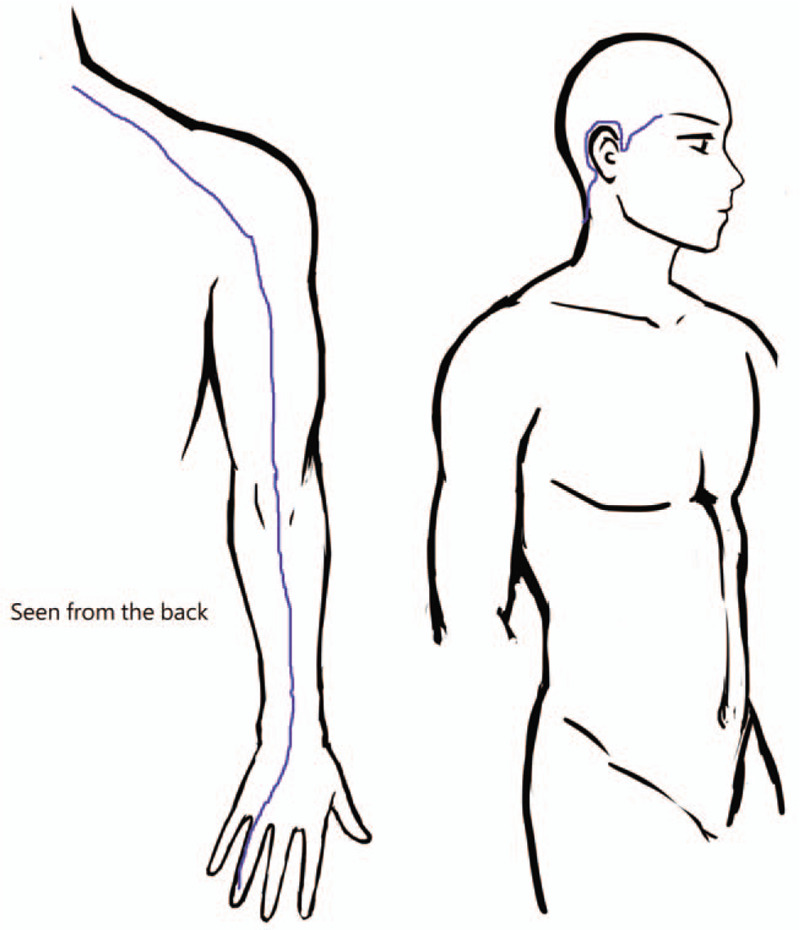
Brief illustration of Hand Shaoyang meridian (=Triple Energizer Meridian, TE meridian).

The fifth limitation is that we did not know the acupoints chosen by the doctors. According to traditional Chinese medicine theories, migraine is considered a disorder of the Shaoyang meridians (including Foot Shaoyang and Hand Shaoyang meridians, Figs. 2 and 3).^[53–56]^ One randomized controlled trial in China also showed that the best treatment effect was achieved by choosing acupoints on the Shaoyang meridian, in comparison with other meridians or sham acupuncture.^[57]^

## 5 Conclusions

In migraine patients who underwent acupuncture treatment, the medical expenditures on emergency care (*P* = .01) and hospitalization (*P* = .01) were significantly lower than patients without acupuncture treatment. For the health policy maker, it is cost effective to encourage combining acupuncture and western medicine to treat migraine patients. For the doctors in routine clinical practice, who may consider to consult acupuncture doctors to deal with the migraine patients together.

## Acknowledgments

The authors would like to thank Enago (www.enago.tw) for the English language review, and Yu-Hsin Chen for drawing the Figures 2 and 3 (foot and hand shaoyang meridians).

## Author contributions

Conceptualization, Sheng-Ta Tsai; Data curation, Mei-Chen Lin; Formal analysis, Mei-Chen Lin; Project administration, Hung-Yu Huang and Yi-Wen Lin; Resources, Hsien-Yin Liao and Chon-Haw Tsai; Supervision, Hung-Yu Huang and Yi-Wen Lin; Validation, Chun-Hung Tseng; Writing – original draft, Sheng-Ta Tsai; Writing – review & editing, Boon-Khai Teoh and San Shao.
